# Occupational accidents in healthcare workers: a bibliometric analysis in Scopus database 2010-2019

**DOI:** 10.47626/1679-4435-2022-724

**Published:** 2023-02-13

**Authors:** Liliana Cruz-Ausejo, Víctor Juan Vera-Ponce, Jenny Raquel Torres-Malca, Juan Carlos Roque-Quesada

**Affiliations:** 1 Universidad Científica del Sur, Facultad de Ciencias de la Salud, Lima, Lima, Peru; 2 Instituto de investigaciones en Ciencias Biomédicas, Universidad Ricardo Palma, Lima, Lima, Peru; 3 Universidad Tecnológica del Perú, Psicología, Lima, Lima, Peru

**Keywords:** occupational accident, healthcare worker, bibliometrics, accidente laboral, personal de salud, bibliometría

## Abstract

Scientific publications in the occupational area have a growing trend towards management of safety in the workplace, despite lack of knowledge on the distribution and characteristics of scientific evidence on occupational accidents in healthcare professionals. This study aims to determine the characteristics and collaboration networks of publications, the co-occurrence of terms, and the main journals on occupational accidents in healthcare professionals among publications indexed in Scopus from 2010 to 2019. This is an observational, cross-sectional, bibliometric study based on publications indexed in the Scopus database. The indicators were number of publications per year, main journals of publication and its quality index, collaboration networks between authors, and co-occurrence of terms. The predominant language of publication was English, the main type of study was the observational one, and nursing professionals represent the main group of interest (31.14% of the articles), contrary to radiologists and/or physical therapists (4% each). The main source of publication about occupational accidents was *Workplace Health and Safety*, and the main themes of investigation were related to puncture injuries and infection by hepatitis B and C. There is a growing trend towards research on occupational accidents of independent authorship, despite the creation of collaboration networks in the last years. Furthermore, nurses and surgeons are the target group of greatest interest, and the main topics cover infectious diseases.

## Abstract (es)

Las publicaciones científicas en el área ocupacional tienen una creciente tendencia en el manejo de la seguridad en el entorno laboral; no obstante, se desconocen la distribución y las características de la evidencia científica sobre accidentes laborales en los profesionales de salud. Este estudio tiene como objetivo determinar las características, redes de colaboración, la co-ocurrencia de términos, y las principales revistas científicas de publicación en accidentes ocupacionales en personal de salud indizadas en Scopus desde 2010 a 2019. Este es un estudio observacional, transversal, bibliométrico a partir de publicaciones indizadas en la base de datos Scopus. Los indicadores fueron número de publicaciones por año, principales revistas de publicación y su índice de calidad, redes de colaboración de autores y co-ocurrencia de palabras. El idioma de publicación predominante fue el inglés, el principal tipo de estudio fue el observacional, y los enfermeros representaron el principal grupo de interés (31,14% de los artículos), al contrario de los radiólogos y/o terapeutas físicos (4% cada uno). La principal fuente de publicación sobre accidentes ocupacionales fue la revista *Workplace Health and Safety*, y los principales temas de investigación se relacionan a lesiones punzocortantes y contagios por hepatitis B y C. Se muestra una creciente tendencia en investigación sobre accidentes ocupacionales de autoría independiente, a pesar de la formación de redes en los últimos años. Además, enfermeras y médicos cirujanos son el grupo objetivo de mayor interés, y los tópicos principales abarcan enfermedades infecciosas.

## INTRODUCTION

Occupational accidents represent a health, economic and social issue that results in monetary losses and psychosocial consequences. Their cause may be explained by two theories, one based on human errors and the other on system errors, one independent from the other; however, accidents in the workplace may be better explained by the interaction of both.^1,2^

Healthcare workers are continuously exposed to occupational risks that may cause occupational accidents and injuries.^3^ Professionals under training usually suffer occupational accidents. A previous study revealed that 80.7% of these accidents are caused by an agent of known source, that 76.7% involve blood, and that the 16.2% of serological source was positive for hepatitis C, and 10.9% for HIV; whereas other study showed that 91.1% are caused by needle sticks.^4,5^ With regard to the nature of these biological accidents, 52.6% are caused by percutaneous injuries to the hands, and 33.5% to the face and neck.^6^ Additionally, nurses (17.3%) and students (67.3%) aged 16-30 years are the most frequently injured professionals, especially in emergency services.^5,7^

In the same sense, musculoskeletal injuries resulting from patient transfer or prolonged hours in operating rooms cause work incapacity, leading to paid leave of absence.^8^ Furthermore, assistant technicians and therapists who perform more than 4 patient transfers per day and from 5 to 8 transfers have a 2.58-fold and a 6.6-fold higher likelihood of suffering back injury, respectively, compared with those who perform only 1 patient transfer per day.^8,9^

Moreover, injuries from ionizing radiation (IR) have gained relevance. Recently, a higher prevalence of subcapsular cataract was reported in interventional cardiologists exposed to IR,^10^ whereas radiologists exposed to a low dose of IR have a hazard ratio of cataract of 1.25 (95% confidence interval: 1.06-1.47), associated with factors such as diabetes mellitus and hypertension.^11^ Other occupational accidents, in turn, involve fainting and physical violence.^12^

Scientific publications in the occupational field have a growing trend towards management of safety in the workplace, evolving from musculoskeletal aspects such as back pain towards psychosocial aspects and population quality of life.^13^ Particularly in the health care sector, the interest has shifted from mental health issues^14-17^ to hospital infection^18^ and resource management,^19^ which leads to the creation of collaboration networks on the theme of infectious diseases such as tuberculosis and influenza.^20-22^

However, the distribution and the characteristics of scientific evidence on occupational accidents in these professionals are unknown. Therefore, the aim of this study is to quantify the scientific productivity concerning studies about occupational accidents in healthcare workers published in Scopus from 2010 to 2019, through productivity and collaboration indicators. The results intend to describe trends in the scientific production on occupational health to promote the development of sustainable practices and studies.

## METHODS

### STUDY DESIGN AND SOURCE OF INFORMATION

An observational, descriptive study was conducted through a bibliometric analysis of publications from 2010 to 2019 found in Scopus. This database was selected due to its diversity, since it has more than 21,000 indexed scientific journals and follows a rigorous selection process, making it one of the main sources of information for bibliometric studies.^23^

### SEARCH STRATEGY

A search algorithm for use in Scopus was developed in September 2020, applying the terms “occupational accident”, “work-related accident”, “healthcare personnel”, “healthcare worker*.” Different designations were used for the health care professionals of each area, such as: “emergency medical technician,” “surgeon,” “laboratory,” “technician,” “radiologist,” etc. Furthermore, the search was limited using the terms PUBYEAR, LIMIT-TO (SRCTYPE, PUBSTAGE, DOCTYPE) for the period of publication, status of publication, and type of document (articles), respectively.

The adequacy of the search strategy was assessed by two authors (LCA and VJVP), who developed independent algorithms, which were compared to obtain the final strategy. A third author (JRTM) performed an independent search in order to corroborate the inclusion of articles on the theme in the final algorithm. The search was performed on a single day: September 10^th^, 2020.

### ANALYSIS OF RESULTS

The search yielded 820 results. Subsequently, LCA and JCRQ performed the quantitative and qualitative review of the studies, for which a data extraction form was created in Excel 2010® including: title of the document, year of publication, language, professional group of interest, and type of study. Inclusion criteria consisted of studies addressing accidents or injuries produced in a work environment. The forms were compared; in case of divergence, the article was fully reviewed and disagreement was solved by consensus. Finally, 289 articles were retrieved.

These data were downloaded in the bib. format and analyzed using the Bibliometrix R-package (https://bibliometrix.org/). The following characteristics of the studies were described: language, type of study, group of interest analyzed, and publications by year. Furthermore, the ten main sources of publication were described, including number of articles and journal’s quality index.

The collaboration networks between authors and the analysis of co-occurrence of the terms included in the title and abstract of publications were presented as network visualization maps using the VOSviewer, version 16.6.^24^

### ETHICS IN INVESTIGATION

The records were downloaded from Scopus, as secondary data, with no contact or interaction with human beings. There were no questions on the ethical aspects of the study, and no approval from an ethics committee was required.

## RESULTS

The systematic search yielded 289 articles about occupational accidents in healthcare workers from 2010 to 2019. As shown in Table 1, the full text of the articles selected was published in 7 different languages. English was the predominant language of the studies, with 254 articles (87.89%). Of the articles written in other languages, eight (2.77%) were written in German, followed by six (2.77%) in Spanish, and five in Portuguese (1.73%). A predominance of observational articles was identified, including qualitative and ecological studies, which involve health workers of different areas. The predominant group of interest was nursing professionals (31%), considering their three types (licensed nurses, nursing students, and nursing technicians), followed by physicians and surgeons.

**Table 1:** Characteristics of studies on occupational accidents in the healthcare workers published in Scopus 2010-2019

Characteristics of the studies (n = 289)	n (%)
Language	
English	254 (87.89%)
German	8 (2.77%)
Spanish	6 (2.07%)
Portuguese	5 (1.73%)
Turkish	5 (1.73%)
Italian	4 (1.38%)
French	4 (1.38%)
Other	3 (1.05%)
Type of studies	
Primary studies	
Observational	278 (96.19%)
Experimental	5 (1.73%)
Secondary studies	6 (2.08%)
Group of interest studied	
Nursing professionals (licensed nurses, nursing students, nursing technicians)	90 (31.14%)
Physicians (residents, specialists)	36 (12.45%)
Surgeons	34 (11.77%)
Emergency personnel (technicians, nurses, medical technologists)	12 (4.16%)
Dental professionals (including assistant technicians)	21 (7.27%)
Radiologists	4 (1.38%)
Physical therapists	4 (1.38%)
Healthcare workers in general	88 (30.45%)

### TEMPORAL TRENDS

**Figure 1:**
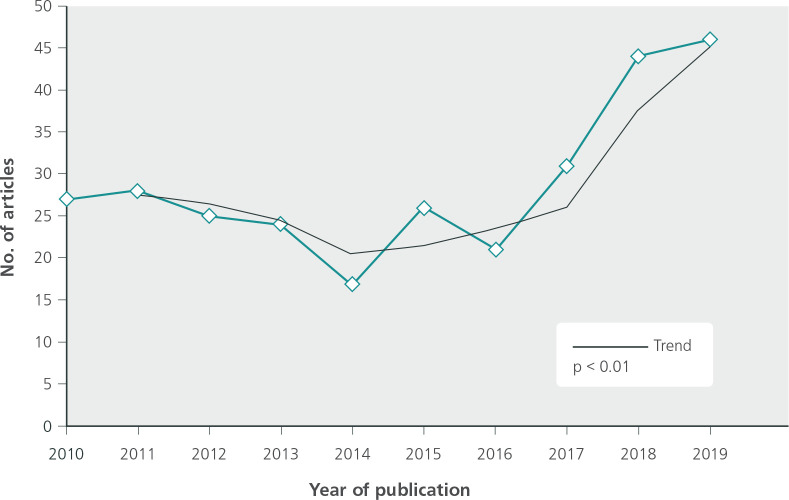
Scientific annual production about occupational accidents in health care workers, Scopus 2010-2019.

With regard to the number of publications, there was a regularity in the number of articles published in the first four years assessed, a decrease from 2014 to 2016, and then a significant increase (p < 0.001) in the last four years assessed (Figure 1). The greatest percentage of growth (29%) was observed between 2017 and 2018, and the peak of publications was observed in 2019, with 46 articles. This shows a growing trend during the last years, considering the year 2016 as the cutoff point for this trend.

### MAIN JOURNAL OF INTEREST

Table 2 provides information of the 10 more active journals regarding publications about occupational accidents in healthcare workers. These 10 journals published 18.6% of the articles (n = 54/289), and the remaining articles were published in 151 other journals.

**Table 2:** Journals with the highest number of publications on occupational accidents in healthcare workers, Scopus 2010-2019

No. in the ranking	Journal	No. of articles	% of articles	SJR 2019^*^	Quartile
1	*Workplace Health and Safety*	11	3.81%	0.369	Q3
2	*American Journal of Industrial Medicine*	8	2.77%	0.65	Q2
3	*Infection control and Hospital Epidemiology*	5	1.73%	1.555	Q1
4	*International Journal of Environmental Research and Public Health*	5	1.73%	0.739	Q2
5	*Work*	5	1.73%	0.596	Q2
6	*American Journal of Infection Control*	4	1.38%	0.989	Q1
7	*International Archives of Occupational and Environmental Health*	4	1.38%	0.768	Q2
8	*Plos One*	4	1.38%	1.023	Q2
9	*Revista Brasileira de Medicina do Trabalho*	4	1.38%	0.197	Q4
10	*Techniques in Vascular and Interventional Radiology*	4	1.38%	0.445	Q3

* SCImago Journal Ranking 2019

The journal with the highest number of published articles was *Workplace Health and Safety,* which had a SCImago Journal Rank (a measure of journal’s impact, influence, and prestige calculated from the average number of weighted citations received by documents published in the journal in the previous 3 years) of 0.369 for the year 2019 and quartile Q3, followed by the *American Journal of Industrial Medicine*, whose quality index of the publications was 0.65. Taken together, these two journals account for 6.6% of the total of publications in the topic of interest.

### TOPICS OF PUBLICATION

**Figure 2:**
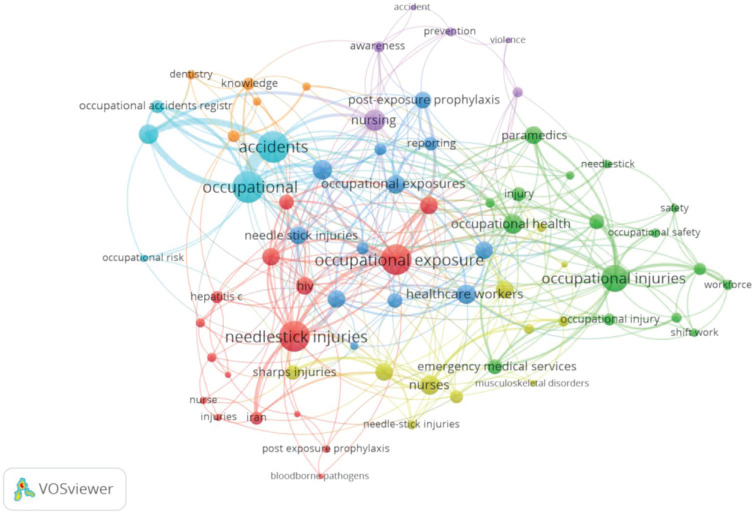
Co-occurrence analysis of terms in titles and abstracts using VOSviewer.

The co-occurrence network of terms in titles and abstracts were identified (Figure 2). The lower threshold number of terms considered was 3, and weight was assigned according to the occurrence of the terms. The graph showed the terms grouped into 7 clusters: 1) hepatitis C, needle stick accidents; 2) accidents in emergency services; 3) infection by pathogens due to contact with fluids: blood; 4) musculoskeletal injuries in nurses, paramedics, and in the operating room; 5) accidents that imply violence; 6) accidents due to exposure to radiation; and, finally 7) accidents with dental professionals and exposure to hepatitis B.

### COLLABORATOR NETWORKS BETWEEN AUTHORS

**Figure 3:**
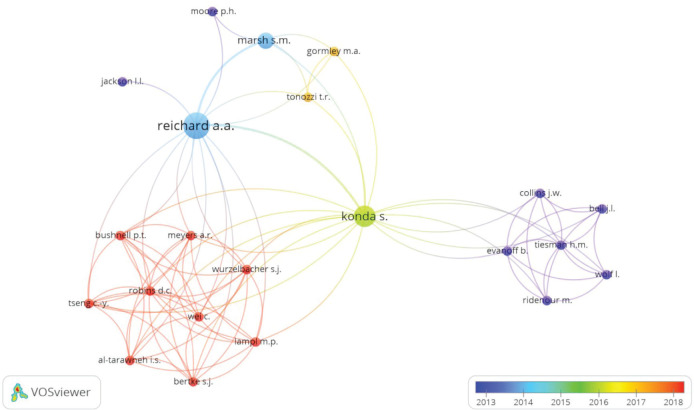
Co-authorship analysis using VOSviewer for publications on occupational accidents in healthcare workers.

Co-authorship analysis (Figure 3) included 1,208 authors, of which 22 worked in collaboration networks and were grouped into 3 clusters, of which Konda and Reichard are the main collaborators, and the weight was assigned according to the number of publications per year. The graph showed the creation of a greater network in 2018, including Meyer, Bushnel, Robins, Bertke, Wei, among other authors with recent publications. The graph excluded authors who presented only one publication.

## DISCUSSION

Occupational accidents in healthcare workers are a challenge for the health system, making research in this field a subject of great interest. This was evidenced by the significant and constant shift in the number of publications from 2016 to 2019 (p < 0.001). A study on occupational health, although with a wider theme, showed a growing trend from 1992 to 2018, in line with the trend observed in this study for 2010-2019, and this sustainable growth is likely to continue up to 2027.^22^ Observational studies, in turn, especially cross-sectional ones (case reports) gain greater importance in demonstrating accidents.

The most studied professional group was nursing professionals, including licensed nurses, nursing technicians, and nursing students; the most addressed theme in this group was biological accidents with blood fluids and infections by virus such as hepatitis B and C. Previous studies reported that this occupational group is the most affected by puncture accidents,^5,7^ which could be closely related to its role in the operating room and in the emergency service, where these professionals are exposed to high levels of occupational stress, which affects concentration and manual dexterity, leading to accidents.^25,26^

During the study period, the main journals with publications on the theme were *Workplace Health and Safety* and the *American Journal of Industrial Medicine*, with 11 and 8 articles and a quartile Q3 and Q2, respectively. The first one is the official publication of the American Board for Occupational Health Nurses and is primarily focused on worker productivity, safety, and management, which would highlight the importance of this journal in the area and make it the first-choice journal to report occupational accidents and injuries.^27^

Additionally, concerning the main theme of publications, there is evidence of interest for topics on infectious diseases, such as infection by hepatitis B and C, the result of injuries and/or contact with biological material in nurses, physicians, and dental professionals, findings consistent with those of previous studies on infectious diseases and internal medicine.^4-7,22^ Conversely, back and upper limb musculoskeletal injuries in paramedics and physical therapists is still a pivotal theme, although the number of investigations was not significant in the second.^8,9^ This could be due to the fact that accidents occur mainly in emergency services and operating rooms, thus preventing the timely report of these findings, which reminds of the need for legislating laws to protect healthcare professionals.^28^

Finally, our study suggests that there was the creation of authorship collaboration networks from 2013, period when collaborations in the theme started to be observed, to 2016. We also showed the connection of collaboration networks by authors such as Reichard and Konda, the latter of whom was a recurrent collaborator and the link between several networks. It is worth noting that the remaining authors prefer to work independently, which could be a limitation to obtain funding for the development of high-impact studies.^29,30^

## CONCLUSIONS

This study evaluated publications about occupational accidents indexed in Scopus during 2010-2019. Its findings indicate a growing trend towards research with independent authorship, despite the creation of collaboration networks in the last 6 years. It was also observed that the themes covered puncture injuries and exposure to biocontaminated fluids, especially in nurses and surgeons working in operating rooms and emergency services, the places with the highest incidence of accidents. Other professional groups less explored were radiologists and physical therapists, despite evidence on accidents due to exposure to IR and patient transfer, respectively.
